# Evaluation of Six Weekly Oral Fecal Microbiota Transplants in People with HIV

**DOI:** 10.20411/pai.v5i1.388

**Published:** 2020-12-30

**Authors:** Netanya S. Utay, Ana N. Monczor, Anoma Somasunderam, Sofia Lupo, Zhi-Dong Jiang, Ashley S. Alexander, Malcolm Finkelman, Karen J. Vigil, Jordan E. Lake, Blake Hanson, Herbert L. DuPont, Roberto C. Arduino

**Affiliations:** 1 Division of General Medicine, Department of Internal Medicine, McGovern Medical School at The University of Texas Health Science Center at Houston, Houston, Texas; 2 Kelsey Research Foundation, Houston, Texas; 3 Division of Infectious Diseases, Department of Internal Medicine, McGovern Medical School at The University of Texas Health Science Center at Houston, Houston, Texas; 4 School of Public Health at The University of Texas Health Science Center at Houston, Houston, Texas; 5 Associates of Cape Cod Inc., Falmouth, Massachusetts

**Keywords:** HIV, microbiome, fecal microbiota transplant, inflammation

## Abstract

**Background:**

Reduced microbiota diversity (dysbiosis) in people with HIV (PWH) likely contributes to inflammation, a driver of morbidity and mortality. We aimed to evaluate the safety and tolerability of 6 weekly oral fecal microbiota transplants (FMT) administered to reverse this dysbiosis.

**Methods:**

Six PWH on suppressive antiretroviral therapy (ART) received 6 weekly doses of lyophilized fecal microbiota product from healthy donors. Shotgun sequencing on stool before, after last FMT, and 20 weeks thereafter was performed. Inflammation and gut permeability biomarkers were measured.

**Results:**

Median age at week 0 was 39 years, CD4^+^ T cell count 496 cells/mm^3^, HIV RNA levels <20 copies/mL. FMT was safe and well-tolerated. α diversity increased in 4 participants from weeks 0 to 6, including the 3 with the lowest α diversity at week 0. At week 26, α diversity more closely resembled week 0 than week 6 in these 4 participants. Metagenomic analysis showed no consistent changes across all participants. One participant had high gut permeability and inflammation biomarker levels and low α diversity that improved between weeks 0 and 6 with a shift in distribution.

**Conclusions:**

Weekly FMT was safe and well-tolerated. α diversity increased in participants with the lowest baseline α diversity during the treatment period. Future randomized, controlled trials of FMT should consider evaluating PWH with greater inflammation, gut damage, or dysbiosis as this population may be most likely to show a significant response.

ClinicalTrials.gov Identifier: NCT03329560

## INTRODUCTION

People with HIV (PWH) taking suppressive antiretroviral therapy (ART) remain at increased risk of cardiovascular events, malignancies, and other comorbidities, likely due to persistent inflammation [1]. A key driver of this inflammation is increased translocation of microbial products across a permeable gut barrier [2]. In HIV, CD4^+^ T cells are rapidly depleted from the intestinal epithelial barrier, with destruction of tight junctions, enterocyte death, decreased IgA and mucus production, and dysfunctional macrophages. Levels of biomarkers of increased gut permeability, microbial translocation, and systemic inflammation remain increased despite the long-term treatment of HIV infection, and these biomarkers predict morbidity and mortality [3–5].

Many studies have demonstrated that PWH have an abnormal gut microbiome, characterized by decreased α diversity (dysbiosis) and a shift toward more pro-inflammatory *Proteobacteria* and fewer anti-inflammatory bacteria that produce short chain fatty acids (SCFAs) [6]. *Erysipelotrichaceae, Enterobacteriaceae, Desulfovibrionaceae*, and *Fusobacteria* are increased, whereas *Lachnospiraceae, Ruminococceae, Bacteroides*, and *Rikenellaceae* are decreased in the microbiomes of PWH. This abnormal microbiome may perpetuate gut damage and chronic systemic inflammation [7]. Some studies have found that alterations in gut flora, particularly increases in *Prevotella* and decreases in *Bacteroides*, only affect men who have sex with men (MSM) [8–11]. However, *Proteobacteria, Desulfovibrio, Enterobacteriaceae, Fusobacteria, Ruminococcaceae*, and *Lachnospiraceae* do not seem to differ between MSM and non-MSM participants [6]. Nonetheless, these findings suggest that sexual activity needs to be considered when conducting microbiome studies.

Fecal microbiota transplant (FMT) is an emerging treatment modality and is in the guidelines for treatment of refractory *Clostridioides difficile* infection [12]. FMT is being explored for inflammatory bowel disease (IBD), Alzheimer's disease, non-alcoholic fatty liver disease, and other conditions. A one-time FMT delivered by colonoscopy was safe in 6 PWH and showed minimal shifts in the microbiota [13]. However, in other chronic conditions such as IBD, multiple FMTs may be more effective [14,15].

We developed an oral, lyophilized, encapsulated FMT product derived from stool of healthy donors [16]. This product has 84% efficacy in treating refractory *C. difficile* infection with excellent tolerability. We hypothesized that 6 weekly FMTs with this lyophilized product delivered by the oral route would be safe, increase α diversity (i.e., decrease dysbiosis) and shift the microbiome in 6 MSM with HIV on suppressive ART.

## METHODS

### Study Design

Participants were eligible if they were MSM, HIV positive, on continuous ART for ≥24 weeks, with CD4^+^ T cell count >350 cells/mm^3^, HIV RNA levels <20 copies/ml for ≥12 weeks, and absolute neutrophil count ≥1000 cells/mm^3^. Participants were excluded if they commenced ART during acute or early HIV infection; had hepatitis B or C (detectable HCV RNA level) co-infection; received antibiotics, investigational therapies, or vaccines within 60 days; had serious illness within 30 days, cirrhosis, chronic gastrointestinal disease, diabetes mellitus, acute or persistent diarrhea within 60 days; or used immunosuppressive drugs, immune modulators, antineoplastic agents, prebiotics or probiotics for >3 consecutive days within 60 days. We recruited 6 participants from Thomas Street Health Center in Houston, Texas. This study was reviewed and approved by the UTHealth Committee for the Protection of Human Subjects. All participants signed an informed consent form. Human experimentation guidelines of the United States Department of Health and Human Services and UTHealth were followed in the conduct of clinical research. ClinicalTrials.gov Identifier: NCT03329560.

### Study Intervention

The encapsulated lyophilized FMT product, PRIM-DJ2727, was prepared as previously described [16]. Donors were healthy men age 56 to 68 years old with BMI <30 kg/m^2^ and no personal or family history of disease suspected to be transmitted by the microbiome. Each participant received the investigational product derived from 150 grams of stool (lyophilized to approximately 2.25 g) from 1 of 3 donors, weekly for 6 weeks. Participants fasted except for water for 8 hours before FMT. They took the product under supervision and were monitored for 1.5 hours after ingestion. Serum and EDTA plasma and stool by self-collected rectal FLOQSwabs were obtained from the participants. Transient elastography with controlled attenuation parameter (Fibroscan®) was performed at weeks 0 and 26.

### Isolation of DNA from Stool

DNA from the stool samples was isolated using the QIAamp Biostic Bacteremia DNA kit (Qiagen, Germantown, MD). DNA concentration was determined using Nanodrop (Thermo Scientific, Waltham, MA).

### Biomarker Measurements

Biomarkers were measured on EDTA plasma or serum. Intestinal fatty acid binding protein (I-FABP) was measured using the DuoSet ELISA Kit; sCD14 and IL-6 were measured by ELISA using Quantikine kits; sCD163 and sTNFRII were measured using Luminex high-performance assays (all R&D systems, Minneapolis MN). Serum (1,3)-β-D-glucan was measured using the Fungitell^®^ kit (Associates of Cape Cod, Inc., Falmouth, MA). Other inflammation biomarkers were measured using the Luminex platform (Bio-Plex Pro™ Human Inflammation Panel 1, Bio-Rad Laboratories, Inc., Hercules, CA).

### Sequencing

DNA was analyzed by shotgun sequencing to achieve strain level resolution of the microbiome (CosmosID, Rockville, MD). Fragment libraries were generated for the DNA using the ThermoFisher IonXpress Plus Fragment Library kit according to the manufacturer's instructions. The libraries were sequenced on an Ion S5XL sequencer to generate 200bp sequences. The depth of the reads in the samples averaged 22.9M, with a standard deviation of 6.3M, a range of 15.8-35.3M, and a median of 21.1M reads. 546 bacterial species and 765 bacterial strains were identified with high confidence. These organisms met a filtering threshold that is based on internal statistical scores determined by analyzing a large number of diverse metagenomes [17].

### Metagenomic Profiling

Unassembled sequencing reads were directly analyzed by the CosmosID bioinformatics platform (CosmosID Inc., Rockville, MD), as previously described [18–21] for multi-kingdom microbiome analysis and quantification of an organism's relative abundance. The system utilizes curated genome databases and a high-performance data-mining algorithm that rapidly disambiguates hundreds of millions of metagenomic sequence reads into the discrete microorganisms engendering the particular sequences.

### Functional Analysis

For functional analysis of the metagenomic data, paired-end reads of the sequencing data were trimmed using BBDuk (https://jgi.doe.gov/data-and-tools/bbtools/) with the parameters minlen=25 qtrim=rl trimq=20. MegaHit [22] was used to construct assemblies from filtered raw reads of metagenomes using 77, 99, 127 values for K-mer size parameter. Each assembled metagenome was sent to Prokka [23] to predict ORF-based protein coding genes and assign functions to the predicted genes in text and GenBank formats. Sequences of the genes, in Fasta format, were used to map to the raw FastQ files using BBMap with allowed fragments per kilobase of gene per million (FPKM) parameter, which produced sample-wise output in text format. Sequences of predicted protein coding genes, identified in Fasta format from the Prokka, were sent to Inter-ProScan for assignment of KEGG pathways and GO processes, including 22 pathways and 143 processes. FPKM values of every protein/gene assigned to each of the KEGG pathway or GO process were derived from mapping the gene to the FPKM list of the genes identified in the previous step. To obtain abundance of each of the KEGG pathways and GO processes, the FPKM values of genes in a pathway or process were summed up and considered as the abundance of the pathway and process, respectively.

### Statistical Methods

Data between time points were compared using Wilcoxon matched-pairs signed rank test and between PWH and donors using Student's t test. Spearman correlation coefficient was used for correlations using GraphPad Prism version 6.2 (San Diego, CA).

## RESULTS

### Baseline Characteristics

Median age was 39 (range 32-56) years, median BMI 27.7 kg/m^2^ (range 25.7-31.8), and median CD4^+^ T cell count 496 (range 393-1029) cells/mm^3^; all participants had HIV RNA levels <20 copies/mL (Table 1). Four participants were Hispanic/Latino, 1 was White non-Hispanic/Latino, and 1 was Black/African American. Median time since diagnosis was 5.5 (range 2-35) years, and median time on ART was 5.5 (range 2-7 years) years. No participant consumed alcohol more than twice a week.

**Table 1.** Baseline Characteristics

	PID1	PID2	PID3	PID4	PID5	PID6
Age (years)	47	40	56	33	37	32
Race	White	White	Black	White	White	White
Ethnicity (Hispanic)	No	Yes	No	Yes	Yes	Yes
CD4 (cells/mm^3^)	1029	505	510	471	393	487
CD8 (cells/mm^3^)	2587	853	857	825	837	548
CD4/CD8	0.29	0.59	0.60	0.57	0.47	0.89
Years since diagnosis	12	4	35	7	3	2
Years on ART^1^	7	4	7	7	3	2
ART regimen	TAF/ FTC/ cEVG^2^	TAF/ FTC/ cEVG	ABC/ 3TC/ DTG^3^	TAF/ FTC + DTG	ABC/ 3TC/ DTG	TAF/ FTC/ cEVG
Weight (kg)	86	93	93	84	77	80
Waist (cm)	99	108	105	110	97	99
Hepatic steatosis (dB/m)	275	280	212	345	298	262

1 ART=Antiretroviral therapy;

2 TAF=Tenofovir alafenamide; FTC=Emtricitabine; cEVG=cobicistat/elvitegravir;

3 ABC=Abacavir; 3TC=Lamivudine; DTG=Dolutegravir

### Safety and Tolerability

Overall, PRIM-DJ2727 was safe and well-tolerated. No participants developed fever or tachycardia. Participant identification (PID)2 developed grade 1 abdominal pain at week 1 and grade 1 nausea at week 2. PID3 had mild bloating on 1 day each in weeks 1, 2, and 4, and moderate bloating on the last 2 days of week 6. No other study drug-related adverse events were noted.

### HIV Parameters

**Figure 1.**
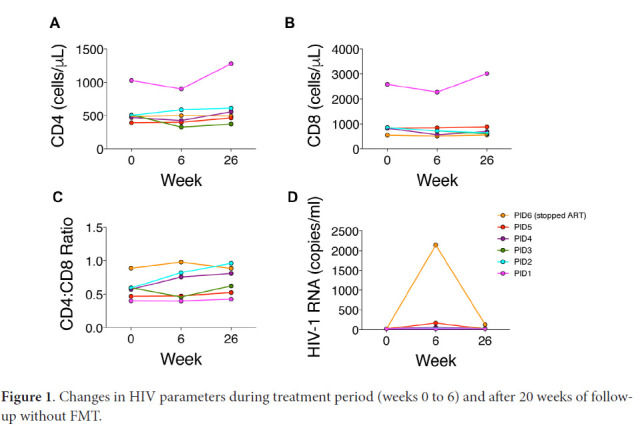
Changes in HIV parameters during treatment period (weeks 0 to 6) and after 20 weeks of follow-up without FMT.

CD4^+^ T cell counts did not change significantly between weeks 0 and 6 (496 [range 393-1029] vs 463 [range 329-900] cells/mm^3^; *P*=0.56) or weeks 6 and 26 (463 [range 329-900] vs 526 [range 329-1279]; *P*=0.13) cells/mm^3^ (Figure 1A). CD8^+^ T cell counts tended to decrease between weeks 0 and 6 (845 [range 548-2587] vs 717 [range 512-2272] cells/mm^3^; *P*=0.06) but did not change significantly between weeks 6 and 26 (717 [range 512-2272] vs 665 [range 558-3014]; *P*=0.56) (Figure 1B). CD4/CD8 ratios did not change significantly between weeks 0 and 6 (0.58 [range 0.40-0.89] vs 0.61 [range 0.40-0.98]; *P*=0.31) or between weeks 6 and 26 (0.61 [range 0.40-0.98] vs 0.71 [range 0.42-0.96]; *P*=0.22) (Figure 1C).

Two participants maintained HIV RNA levels <20 copies/mL at weeks 0, 6, and 26, whereas the other participants developed detectable HIV RNA levels (Figure 1D). Three participants subsequently had undetectable levels. Two of these participants had 34-day gaps between ART refills during the study. PID6 stopped ART at week 6 but subsequently resumed ART with HIV RNA level of 130 copies/mL at week 26.

### Microbiome Effects

**Figure 2.**
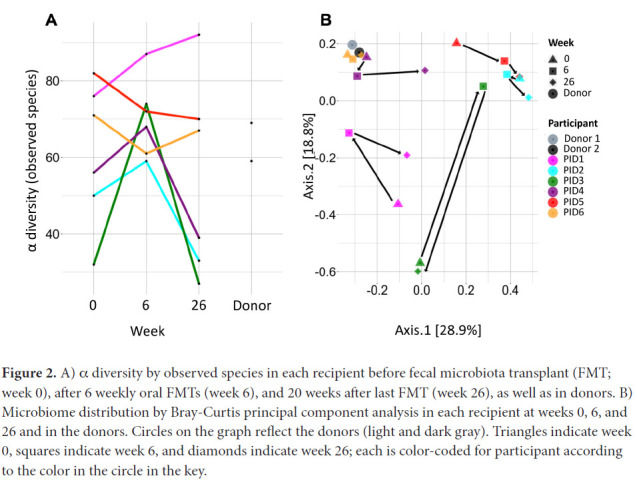
A) α diversity by observed species in each recipient before fecal microbiota transplant (FMT; week 0), after 6 weekly oral FMTs (week 6), and 20 weeks after last FMT (week 26), as well as in donors. B) Microbiome distribution by Bray-Curtis principal component analysis in each recipient at weeks 0, 6, and 26 and in the donors. Circles on the graph reflect the donors (light and dark gray). Triangles indicate week 0, squares indicate week 6, and diamonds indicate week 26; each is color-coded for participant according to the color in the circle in the key.

Mean α diversity by observed species index did not materially change from weeks 0 to 6 (61.2 to 70.2, *P*=0.29; Figure 2A) or from weeks 6 to 26 (70.2 to 52.2, *P*=0.21). α diversity increased in 4 participants (67%) from weeks 0 to 6, including the 3 with the lowest α diversity at week 0 (Figure 2A). At week 26, α diversity more closely resembled week 0 than week 6 in 3 of these 4 participants.

**Figure 3.**
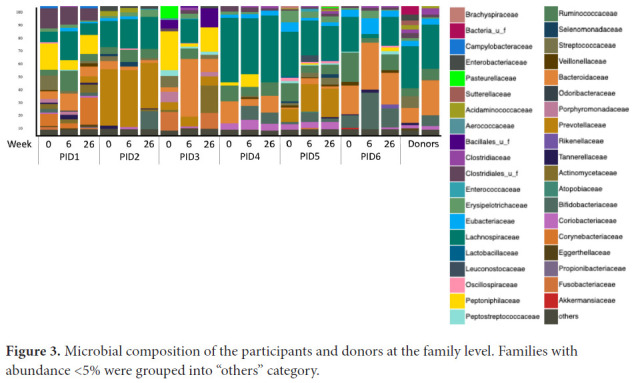
Microbial composition of the participants and donors at the family level. Families with abundance <5% were grouped into “others” category.

Microbiome distribution by Bray-Curtis principal component analysis (PCA) shified towards the donors' distribution in 3 participants (50%) at week 6, all of whom had an increase in α diversity between weeks 0 and 6, but shified away by week 26 (Figure 2B). Microbiome distribution varied across participants (Figure 3). There were no statistically significant changes in any specific strains, species, genera, or families, or in phage distribution.

**Figure 4.**
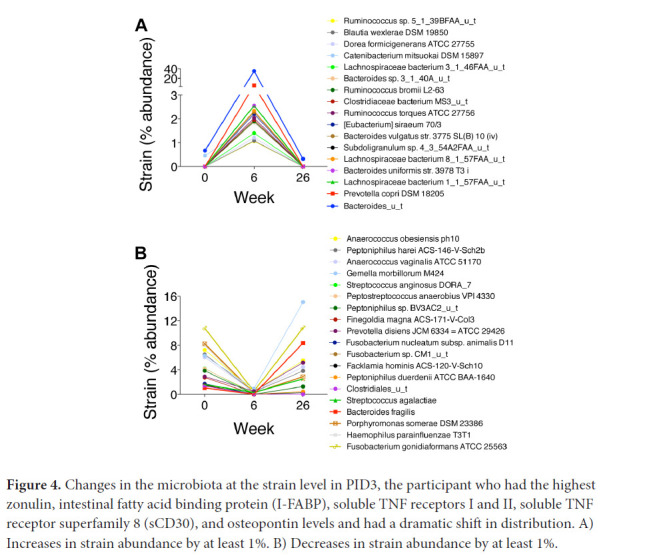
Changes in the microbiota at the strain level in PID3, the participant who had the highest zonulin, intestinal fatty acid binding protein (I-FABP), soluble TNF receptors I and II, soluble TNF receptor superfamily 8 (sCD30), and osteopontin levels and had a dramatic shift in distribution. A) Increases in strain abundance by at least 1%. B) Decreases in strain abundance by at least 1%.

PID3, who had HIV for >35 years and had been taking ART for 7 years, had persistent constipation that resolved between weeks 0 and 6 with a dramatic shift in distribution. Seventeen strains increased by >1% relative abundance (Figure 4A), including several *Bacteroides, Lachnospiraceae*, and *Ruminococcus* strains, all of which synthesize SCFAs and convert primary into secondary bile acids [24–27]. Nineteen strains decreased by >1% relative abundance, including numerous oral microbes (Figure 4B). Of note, PID3 also had increases in numerous bacteriophages, including *Yersinia* phage L-413C, *Enterobacteria* phage P4, and *Escherichia* virus P2, with decreases in *Haemophilus* phage Hp1 (data not shown). His constipation recurred by week 26 with regression of his microbiome to approximate its baseline distribution and α diversity, although the phage changes persisted.

### Metagenomics

Next we evaluated changes in gene expression pathways using metagenomics. Ribosome pathways, reflecting protein synthesis, tended to decrease in participants during the treatment period (*P*=0.09), with decreases in 5 of 6 participants (83%; Supplementary Figure 1A). The 2 donors had higher L-arabinose isomerase activity, reflecting carbohydrate degradation, (Supplementary Figure 1B) and pentose and glucuronate interconversions (Supplementary Figure 1C), than all recipients at all time points. In PID3, several pathways increased between weeks 0 and 6: 1) defense response to Gram-negative bacterium; 2) lipopolysaccharide biosynthesis; 3) spore germination; 4) peptidase activity; 5) porphyrin and chlorophyll metabolism; 6) inositol phosphate metabolism; 7) glycine, serine, and threonine metabolism; and 8) glycerophospholipid metabolism (data not shown). Pathways that decreased during the treatment period in PID3 include 1) cell septum assembly; 2) pathways of energy generation and metabolism (arginine biosynthesis, fumarate metabolic process, iron-sulfur cluster assembly, oxidation-reduction process, ubiquinone activity); 3) urea catabolic process; 4) methionine metabolism; 5) iron ion binding; and 6) quorum sensing (data not shown).

### Markers of Gut Damage and Inflammation

**Figure 5.**
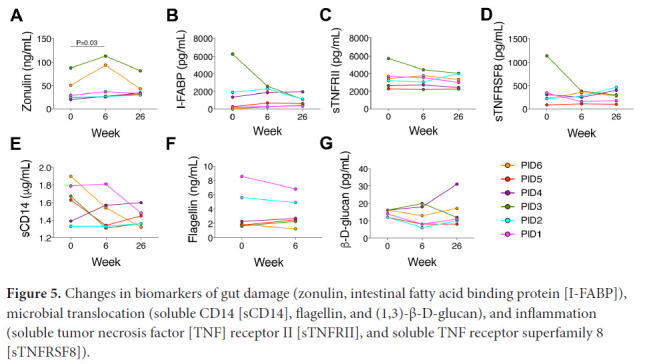
Changes in biomarkers of gut damage (zonulin, intestinal fatty acid binding protein [I-FABP]), microbial translocation (soluble CD14 [sCD14], flagellin, and (1,3)-β-D-glucan), and inflammation (soluble tumor necrosis factor [TNF] receptor II [sTNFRII], and soluble TNF receptor superfamily 8 [sTNFRSF8]).

Next we measured circulating biomarkers of enterocyte turnover, microbial translocation, inflammation, and immune activation (Figure 5 and Supplementary Figure 2). Zonulin increased significantly between weeks 0 and 6: from 27.2 ng/mL at week 0 to 31.7 ng/mL at week 6 (*P*=0.03; Figure 5A), but no other biomarker changed significantly. PID3 had the highest zonulin, I-FABP, sTNFRI, sTNFRII, sTNFRSF8 (sCD30), and osteopontin levels of the participants and among the highest sCD14, sCD163, and (1,3)-β-D-glucan levels but the lowest levels of gp130, IL-6Ra, IL-8, osteocalcin, and TWEAK at week 0 (Figure 5 and Supplementary Figure 2). PID3's I-FABP and sTNFRSF8 levels decreased by >50% between weeks 0 and 6 (Figures 5B and 5D). Of note, higher I-FABP levels tended to correlate with lower α diversity at week 0 (r=-0.77, *P*=0.10) and at all time points combined (r=-0.59, *P*=0.01). Lower α diversity also correlated with lower IFNγ (r=0.97, *P*=0.01) and MMP-1 levels (r=0.85, *P*=0.04) at week 0 and lower IFNγ (r=0.53, *P*=0.02) and MMP-1 (r=0.61, *P*=0.008) and higher sCD30 (r=-0.60, *P*=0.008) and IL-20 (r=-0.52, *P*=0.03) levels at all time points combined.

### Anthropomorphics and Hepatic Steatosis

Weight did not change significantly between weeks 0 and 6 (median 0.5 kilograms [range −5 to 1.6; *P*=0.75) or between weeks 6 and 26 (median 0.7 kilograms [range −0.5 to 1.4; *P*=0.13]). Waist circumference changed by a median of −2.8 cm (range −5.0 to 2.0; *P*=0.19) between weeks 0 and 6 and −0.3 cm (range −4.0 to 3.3; *P*=0.81) between weeks 6 and 26. We found no significant change in hepatic steatosis between weeks 0 and 26 based on transient elastography with controlled attenuation parameter (278 [range 212 to 345] and 276 [range 201 to 300] dB/m, *P*=0.91).

### Confounding Factors

PID2 received amoxicillin for an upper respiratory tract infection and clindamycin and levofloxacin just before week 26 for cellulitis. PID4 switched ART to bictegravir/tenofovir alafenamide/emtricitabine in week 2, as did PID5 and PID6 between weeks 6 and 26. PID6 was also given clindamycin and ceftriaxone for an abscess in week 3.

## DISCUSSION

PWH have increased systemic inflammation, which drives end-organ events. In this pilot study, we administered weekly oral FMT with the goal of assessing safety and tolerability. We evaluated changes in dysbiosis and inflammation during and after the treatment period. We found that 1) oral FMT was safe and well-tolerated in PWH; 2) α diversity increased between weeks 0 and 6 in PWH with low α diversity pre-FMT, but this change did not persist between weeks 6 and 26; 3) microbiome populations shified towards the distribution of the donors between weeks 0 and 6, but no participant had complete engraftment of the FMT; and 4) one participant who had the lowest α diversity and highest I-FABP, zonulin, sTNFRI, sTNFRII, and TNFRSF8 levels at baseline had the greatest shift in microbiome distribution between weeks 0 and 6, with increases in bacteria that produce butyrate and SCFAs, decreases in bacteria of oral origin, and decreases in I-FABP, sCD14, sTNFRI, sTNFRII, TNFRSF8, and IL-6 levels.

The absence of complete engraftment is consistent with findings in other disease states such as *C. difficile* disease and IBD, where there is not complete replacement of the recipient's microbiome with the donor's microbiome, yet clinical benefits and changes in the microbiome with FMT have been observed [28–30]. This suggests that the effects of the microbiome may not be dependent upon a single organism or group of organisms but rather on the functionality of the microbiome. The participant with the greatest dysbiosis had the most significant changes in his microbiome and inflammatory markers, but it cannot be concluded definitively that these changes were due to FMT in the absence of a randomized, controlled trial. Nonetheless, whether inducing dysbiosis such as with antimicrobial agents will facilitate greater engraftment in more recipients is unknown. The development of appropriate metrics of desired functionality is needed so as to better evaluate the influences of microbiome composition and engraftment.

Alternatively, the bacteria and their functionality may be less important than other factors that are also transmitted, such as phages, SCFAs, and bile acids [31,32]. We observed changes in phages in PID3 but no consistent changes overall. SCFAs such as butyrate are crucial energy sources for colonic epithelial cells and tight junction maintenance and therefore critical for gut barrier integrity [33]. SCFAs also modulate neutrophil trafficking, stimulate regulatory CD4^+^ T cell generation, and decrease pro-inflammatory cytokine production by monocytes and macrophages [34]. Gut bacteria such as from *Lachnospiraceae* and *Ruminococcaceae* families convert primary bile acids, originating from the liver, into secondary bile acids [35]. Bile acids, in turn, shape the composition of the gut microbiota. Thus, transmission of secondary bile acids may shape the recipient's microbiome independent of bacteria that are also transmitted.

Levels of (1,3)-β-D-glucan [36], sCD14 [3], and sCD163 [37] were on par with levels observed in people who are HIV-negative, whereas I-FABP and zonulin were consistent with other studies of PWH [3,38]. Together, these findings suggest that while enterocyte and tight junction turnover may be increased, microbial translocation and its ensuing inflammation was minimal in these participants. Thus, these participants may not have needed any intervention. T cell activation, reflected by sTNFRI, sTNFRII, and sTNFRSF8 (sCD30) [39], was high in the participant who had the most dramatic microbiome shift, but these levels declined between weeks 0 and 6. It is tempting to speculate that PWH with high microbial translocation, immune activation, and systemic inflammation at baseline would garner the greatest benefit from FMT, but larger controlled studies of FMT for such a population are needed to determine whether that is the case.

The low-level HIV viremia observed in several participants after starting FMT was unexpected. This may have been due to suboptimal adherence, but raises the question of whether FMT stimulated local inflammation in the gut, where most of the virus resides [40], prompting CD4^+^ T cell activation and HIV RNA production or release from apoptotic cells. The increase in zonulin between weeks 0 and 6 may indicate more tight junction damage or turnover that could be consistent with local inflammation or, alternatively, the increase could reflect improved gut barrier function and less suppression of zonulin production. Systemic inflammation was not increased, and enterocyte turnover did not seem to change based on I-FABP levels. Nonetheless, this transient HIV viremia suggests that HIV RNA levels should be monitored closely in future FMT studies, and gut biopsies may be needed to elucidate the direct effects of FMT on gut integrity and inflammation.

Our study has several limitations. First, the study was small, with numerous confounding factors, and therefore underpowered to detect significant changes in the microbiome. The large number of analyses including the large number of pathways evaluated may have increased the risk of false positive findings in this small sample size. Second, the study lacked a placebo control, so it is challenging to conclude which changes are due to FMT versus which might have happened regardless. Third, the donors were selected based on the safety and efficacy of their stool for treating *C. difficile* patients, which may not translate to efficacy in PWH. In addition, engraftment may be donor-dependent [41]. Fourth, each recipient only received FMT from one donor; combining the microbiota of several healthy people into each FMT may increase the likelihood of a response. Fifth, neither the donors nor the recipients were counseled on a “microbiome-friendly diet” to maximize the likelihood of a healthy microbiome engrafting, and dietary histories were not collected. Lastly, most of the recipients were relatively healthy, with low levels of inflammation biomarkers, and half had α diversity on par with the donors'; therefore, they may not have been the population most likely to benefit. Rather, PWH with increased levels of inflammatory bio-markers and low α diversity may be most likely to benefit from FMT.

## CONCLUSION

In summary, we found that administering multiple oral FMT was safe and well-tolerated. The changes in the microbiome during the treatment phase in some participants encourage us to pursue this line of research with enhanced methods of FMT treatment. Characteristics of an ideal oral FMT product are unknown, as are the optimal dose and treatment schedule. We are now combining the microbiota product of 3 donors, administered twice daily for 12 weeks, to increase the likelihood of significant engraftment in our studies of FMT in other chronic diseases. Whether oral FMT can decrease systemic inflammation in PWH with high baseline inflammation and low α diversity requires larger, controlled studies.

## SUPPLEMENTARY DATA

Supplementary materials are available at the *Pathogens and Immunity* website. Supplementary data may be provided by the authors to benefit the reader. Supplementary data are not copyedited and are the sole responsibility of the authors. Questions or comments related to supplementary materials should be addressed to the corresponding author.
