# Supplementary material for: Evaluation of Six Weekly Oral Fecal Microbiota Transplants in People with HIV
Source: Pathog Immun. 2020 Dec 30;5(1):364–81. doi: 10.20411/pai.v5i1.388 (PMC7815055; doi:10.20411/pai.v5i1.388)
Supplement: Supplementary Figure 1 [file pai-5-364-s01.pdf]

Supplementary Figure 1

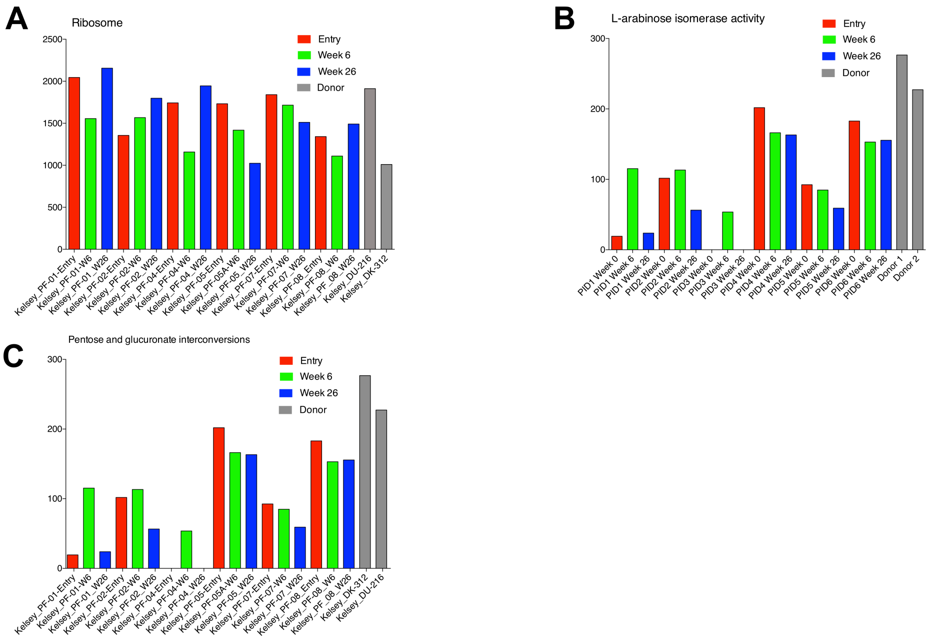

Supplementary Figure 1. Metagenomics analysis. (A) Ribosome pathways tended to decrease during treatment period. (B) L-arabinose isomerase activity was lower in recipients than donors. (C) Pentose and glucuronate interconversions were lower in recipients than donors.
