# Supplementary material for: Evaluation of Six Weekly Oral Fecal Microbiota Transplants in People with HIV
Source: Pathog Immun. 2020 Dec 30;5(1):364–81. doi: 10.20411/pai.v5i1.388 (PMC7815055; doi:10.20411/pai.v5i1.388)
Supplement: Supplementary Figure 2 [file pai-5-364-s02.pdf]

Supplementary Figure 2

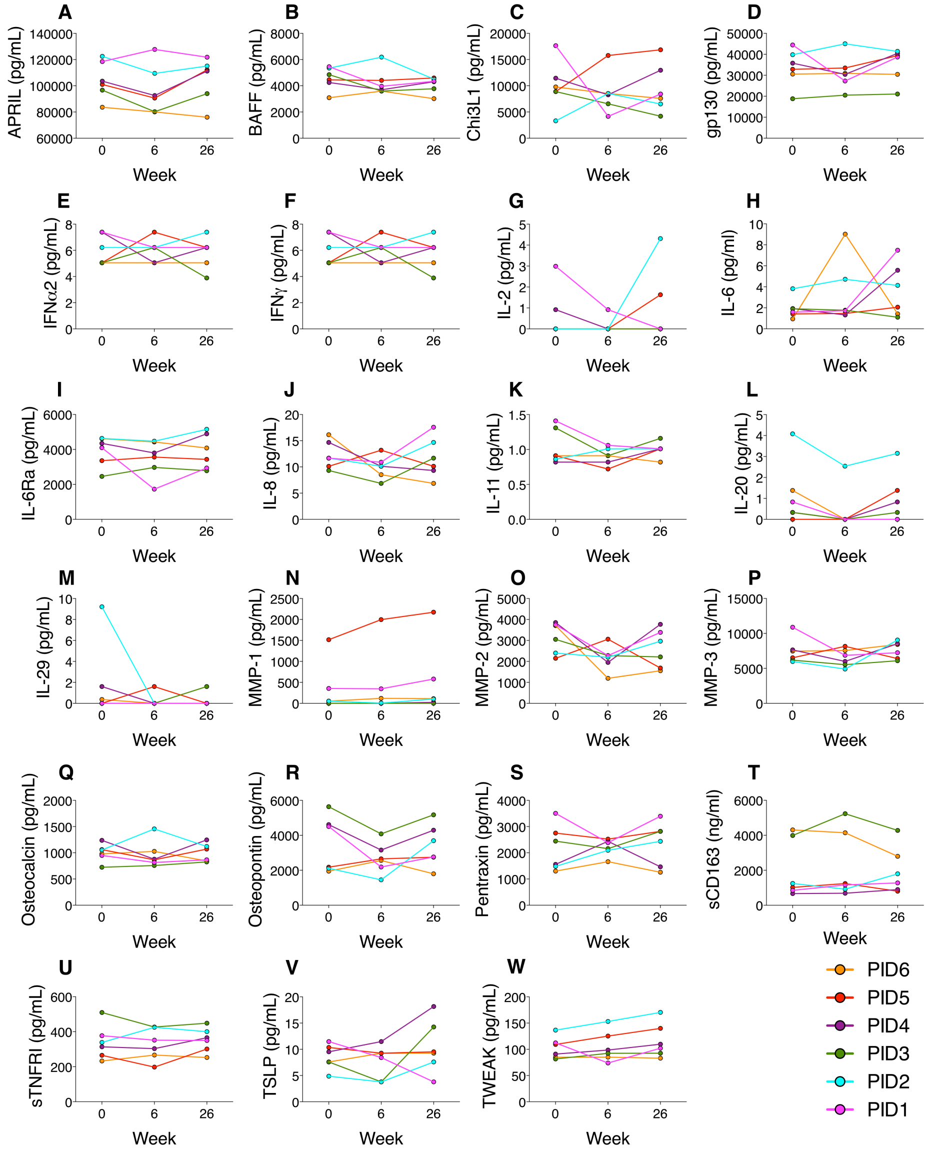

Supplementary Figure 2. Changes in biomarkers of inflammation during treatment period. (A) A proliferation-inducing ligand (APRIL). (B) B-cell activating factor (BAFF). (C) Chitinase 3-Like 1 (Chi3L1). (D) Glycoprotein 130 (gp130). (E) Interferon (IFN) α2. (F) IFNγ. (G) Interleukin (IL) 2. (H) IL-6. (I) IL-6 Receptor (IL-6Ra). (J) IL-8. (K) IL-11. (L) IL-20. (M) IL-29. (N) Matrix metalloproteinase 1 (MMP-1). (O) MMP-2. (P) MMP-3. (Q) Osteocalcin. (R) Osteopontin. (S) Pentraxin. (T) Soluble CD163 (sCD163). (U) Soluble tumor necrosis factor receptor 1. (V) Thymic stromal lymphopoietin (TSLP). (W) TNF-related weak inducer of apoptosis (TWEAK).
